# Third-generation sequencing found LncRNA associated with heat shock protein response to heat stress in *Populus qiongdaoensis* seedlings

**DOI:** 10.1186/s12864-020-06979-z

**Published:** 2020-08-24

**Authors:** Jiahong Xu, Yao Zheng, Shouqin Pu, Xiujie Zhang, Zhihao Li, Jinhui Chen

**Affiliations:** 1 Key Laboratory of Genetics and Germplasm Innovation of Tropical Special Forest Trees and Ornamental Plants, Ministry of Education / Engineering Research Center of Rare and Precious Tree Species in Hainan Province, College of Forestry, Hainan University, Haikou, 570228 P. R. China; 2 Hainan Key Laboratory for Biology of Tropical Ornamental Plant Germplasm, Institute of Tropical Agriculture and Forestry, College of Forestry, Hainan University, Haikou, 570228 P. R. China

**Keywords:** *Populus qiongdaoensis*, Heat stress, Heat shock protein, lncRNAs, miRNAs

## Abstract

**Background:**

As air temperatures increase globally, more and more plants are exposed to heat-stress conditions. Although many studies have explored regulation networks in plants with the aim of improving their heat-stress tolerance, only few have revealed them in trees. Here, individuals of *Populus qiongdaoensis* seedlings, which grows naturally in tropical areas, exposed to heat at 40 °C and the non-coding regulation networks were explored using the PacBio RSII and the Illumina sequencing platform.

**Results:**

In total, we obtained 88,161 full-length transcripts representing 39,343 genes using 5,498,988 long reads and 350,026,252 clean reads, and also 216 microRNAs (miRNAs) via 95,794,107 reads. We then identified 928 putative long non-coding RNAs (lncRNAs), consisting of 828 sense lncRNAs (89.22%), 34 long intergenic non-coding RNAs (3.66%), 16 antisense (1.72%), and 50 sense intronic lncRNAs (5.39%). Under the dual criteria of |log_2_fold-change| ≥ 1 and *P*-value < 0.05, 1690 genes, 25 lncRNAs, and 15 miRNAs were found differentially expressed under the heat stress treatment. Furthermore, 563 and 595 mRNAs were detected as target genes of 14 differently expressed miRNAs and 26 differentially expressed lncRNAs. Functional annotation analysis of these target genes demonstrated they were related to cell membrane stability, plant hormone signal transduction, antioxidation, and aldarate metabolism. Lastly, we uncovered a key interaction network of lncRNAs, miRNAs and mRNAs that consisted of miR1444d, miR482a.1, miR530a, lncHSP18.2, *HSP18.1*, and *HSP18.2*. Expression level analysis showed that miRNAs in the network were up-regulated, while mRNAs and lncRNA were down-regulated, and also found that lncHSP18.2 may *cis*-regulate *HSP18.2*.

**Conclusions:**

Functional enrichment analysis of target genes of miRNAs and lncRNAs indicated that miRNAs and lncRNAs play an important role in the response to heat stress *P. qiongdaoensis*. Lastly, by investigating the miRNA–lncRNA–mRNA network of this species, we revealed that miRNAs may negatively regulate both lncRNAs and mRNAs in tree responses to heat stress, and found that lncHSP18.2 may *cis*-regulate *HSP18.2*.

## Background

In recent years, the growth of plants worldwide has been seriously threatened by frequent high-temperature weather conditions, especially in the tropics [1]. When exposed to high temperatures, plants produce antioxidants [2, 3], phytohormones [4], osmotic adjustment material [5], and heat shock proteins (HSPs) [6], and exhibit decreases in photosynthesis and transpiration [7] as well as cell membrane stability [8]. HSPs are widely studied, including the small heat shock protein (sHSP) identified and characterized from thermotolerant and thermosusceptible cultivars of wheat [9]. Up-regulated expression of heat shock genes and rapid synthesis of new HSPs proteins can enhance plant heat tolerance [10]. For example, *HSP26* is highly expressed under high-temperature stress in *Arabidopsis thaliana* [9], and both *HSP20* and *HSP90* genes are activated in tall fescue (*Festuca arundinacea* Schreb.) grass under high-temperature stress [10]. In chickpea and pigeonpea, Agarwal et al. found that HSP90 gene families in response to heat stress [11]. Further, heterologous expression of the *Trichoderma harzianum HSP70* gene in Arabidopsis increases plant heat tolerance and resistance to other abiotic stresses [12]. In sum, research has confirmed that *HSP* genes play important roles in the responses of plants to high-temperature stress.

In addition to the protein-coding genes, there is increasing evidence that non-coding RNA plays a key role in plants under heat stress, namely in the form of miRNAs and lncRNAs. For example, after 0.5 h of heat stress, birch tree *Betula luminifera* miR160a-3p and miR396b-3p are down-regulated, while miR408a-3p and miR166a-3p are up-regulated, suggesting that these miRNAs mediate the physiological mitigation of heat stress [13]. More recently, lncRNAs were reported to be significantly enriched in loci related to stress tolerance or development in rice [14]. Increasing evidence has revealed that non-coding RNAs can interact and that lncRNA can function as a competitive endogenous RNA to regulate miRNAs [15]. For example, the lncRNA *IPS1* has a binding site for the phosphate starvation-induced miR-399 in Arabidopsis, which would prevent miR-399 from cleaving *IPS1* [16]. Furthermore, because interactions between miRNAs and lncRNAs can trigger the decay of targeted lncRNAs, they could figure prominently in target gene regulation [15]. Although high-temperature stress has been investigated in many plants, most studies have focused on the discovery of differentially expressed genes (DEGs), whereas others have examined the activity of miRNA or lncRNA regulatory genes. In contrast, few studies have examined the effect of high-temperature stress upon the entire miRNA–lncRNA–mRNA network to elucidate the details of plant resistance to this globally important stress factor.

*Populus qiongdaoensis* is a *Populus* that grows in the tropics [17], and it may have a unique heat stress gene response pattern. To provide insight into the molecular regulation mechanisms active in trees in response to heat stress, we used single-molecule real-time (SMRT) sequencing technology to obtain full-length sequences suitable for exploring gene length and lncRNAs [18, 19], and the small RNA sequencing (small RNA-seq) and RNA sequencing (RNA-seq) to reveal the changes in genes, miRNAs, and lncRNAs in *P. qiongdaoensis* after treatment at 40 °C for 1 h. To the best of our knowledge, this study is the first to characterize the transcriptome of *P. qiongdaoensis*; hence it serves as a basis for further research on tree responses to high temperatures and the development of new plant varieties more tolerant of high temperatures.

## Results

### High-quality full-length sequencing provides sufficient sequences for further analysis

**Fig. 1:**
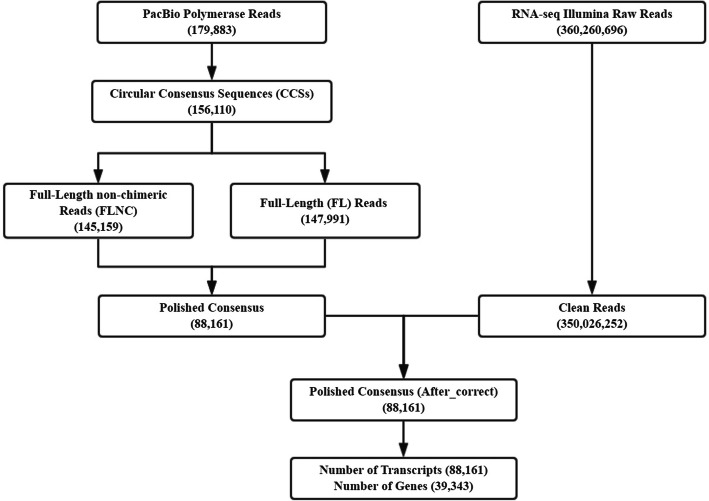
Computational pipeline for the splicing transcripts analysis in *P. qiongdaoensis* by SMRT sequencing and RNA sequencing

To elucidate the heat-responsive mechanism in a tropical tree, the transcriptomes of six seedlings (= samples) of *P. qiongdaoensis* were sequenced and analyzed with the PacBio Sequel platform, a total of 179,883 polymerase reads and 5,498,988 subreads were obtained, with an average read length of 2065 bp (Fig. 1). To provide more accurate sequence information, a circular consensus sequence (CCS) was generated from reads that fully-passed at least twice through the insert, for a total of 156,110 CCSs (average read length of 2531 bp) (Fig. 1). Via SMRTlink detection, 147,991 sequences were distinguished as full-length (i.e., containing the 5′ primer, 3′ primer, and the poly(A) tail) and 145,159 were identified as being full-length non-chimeric (FLNC) reads with low artificial concatemers; mean length of FLNC was 2365 bp (N50 = 2741 bp) (Fig. 1). The FLNC reads with similar sequences were clustered together—using the ICE (iterative isoform-clustering) algorithm—and its consensus sequence is obtained; in this way, non-full-length sequences could then be corrected by the Arrow software, resulting in 88,161 polished consensus sequences (Fig. 1), with a mean length of 2439 bp.

The RNA-seq generated 180,729,720 and 179,530,976 raw reads from the pqq (control) and pqh (heat-stress treatment) samples, with 176,114,318 (pqq) and 173,911,934 (pqh) clean reads remaining after their trimming (Table 1). The raw reads of Illumina sequencing data were then used to correct the SMRT data. After removing redundant and similar sequences, this left a total 101,959,269 nucleotides and 39,343 transcripts with a mean length of 2592 bp. Overall, 39,343 transcripts (corrected isoforms) were subjected to functional annotation by searching the Non-Redundant Protein Database (NR), Nucleotide Sequences (Nt), Swiss-Prot, Gene Ontology (GO), Cluster of Orthologous Groups of proteins (COG), Cluster of Eukaryotic Orthologous Groups (KOG), the protein family (Pfam), and Kyoto Encyclopedia of Genes and Genomes (KEGG) databases, of which a total of 39,113 transcripts (99.42%) were successfully annotated (Supplementary Fig. S1). We analyzed homologous plant species by comparing the transcript sequences to the NR database, which showed that the largest transcripts were distributed in *Populus trichocarpa* (Supplementary Table S1).

**Table 1:** Summary of Illumina sequencing

Library	Groups	Raw reads		Clean reads		Clean bases (G)	
		Total	Average	Total	Average	Total	Average
RNA-seq	pqq	180,729,720	60,243,240	176,114,318	58,704,772.67	26.42	8.81
	pqh	179,530,976	59,843,658.67	173,911,934	57,970,644.67	26.08	8.69
	Total	360,260,696	120,086,898.7	350,026,252	116,675,417.3	52.5	17.5
Small RNA-seq	pqq	53,256,108	17,752,036	51,150,950	17,050,316.67	2.663	0.89
	pqh	45,724,929	15,241,643	44,643,157	14,881,052.33	2.286	0.76
	Total	98,981,037	32,993,679	95,794,107	31,931,369	4.949	1.65

### miRNAs and lncRNAs identified in *P. qiongdaoensis*

Through small RNA-seq, a total of 98,981,037 high-quality reads were obtained from the two sRNA libraries generated from the control (53,256,108) and heat stress (45,724,929) treatments (Table 1). After removing the low-quality reads, 51,150,950 (pqq) and 44,643,157 (pqh) clean reads with lengths of 18–30 nt were obtained respectively from the control and heat-treated samples of sRNA libraries (Table 1). Most sRNAs were 18 to 24 nucleotides in length (Supplementary Fig. S2). In total, we identified 126 (pqq) and 104 (pqh) known miRNAs belonging to 66 miRNA families, amounting to 132 miRNAs (Supplementary Table S2). Additionally, we analyzed the first base of a known miRNA with a length of 18–30 nt, finding that the U battle ratio was largest (Supplementary Fig. S3 and Supplementary Table S3). Similarly, for a total of 21 novel miRNAs predicted in the control and heat-treated groups by small RNA sequencing (Supplementary Table S4), we analyzed the first base of a novel miRNA with a length of 18–30 nt; this also showed that the U battle ratio was largest (Supplementary Fig. S4; Supplementary Table S5).

The clean reads per sample obtained by Illumina sequencing were aligned to reference sequence (*ref*), and read count for each gene obtained from the mapping results. The number of mapped reads in the six RNA-seq libraries ranged from 43,356,404 to 58,597,274, and the proportions mapped ranged from 86.09 to 88.38% (Supplementary Table S6). This process used RSEM software, for which the parameters of the bowtie2 comparison software were set to end-to-end and sensitive modes. For the identification of lncRNA, the PacBio data were used and then filtered through the basic selection and potential coding capability analysis; this predicted the total number of lncRNAs to be 928 (Supplementary Fig. S5). We classified them into four groups: 828 sense lncRNAs (89.22%), 34 lincRNAs (3.66%), 16 antisense (1.72%) and 50 sense intronic lncRNAs (5.39%) (Supplementary Table S7).

### Analysis of differentially expressed mRNAs, lncRNAs, and miRNAs in response to heat stress

**Fig. 2:**
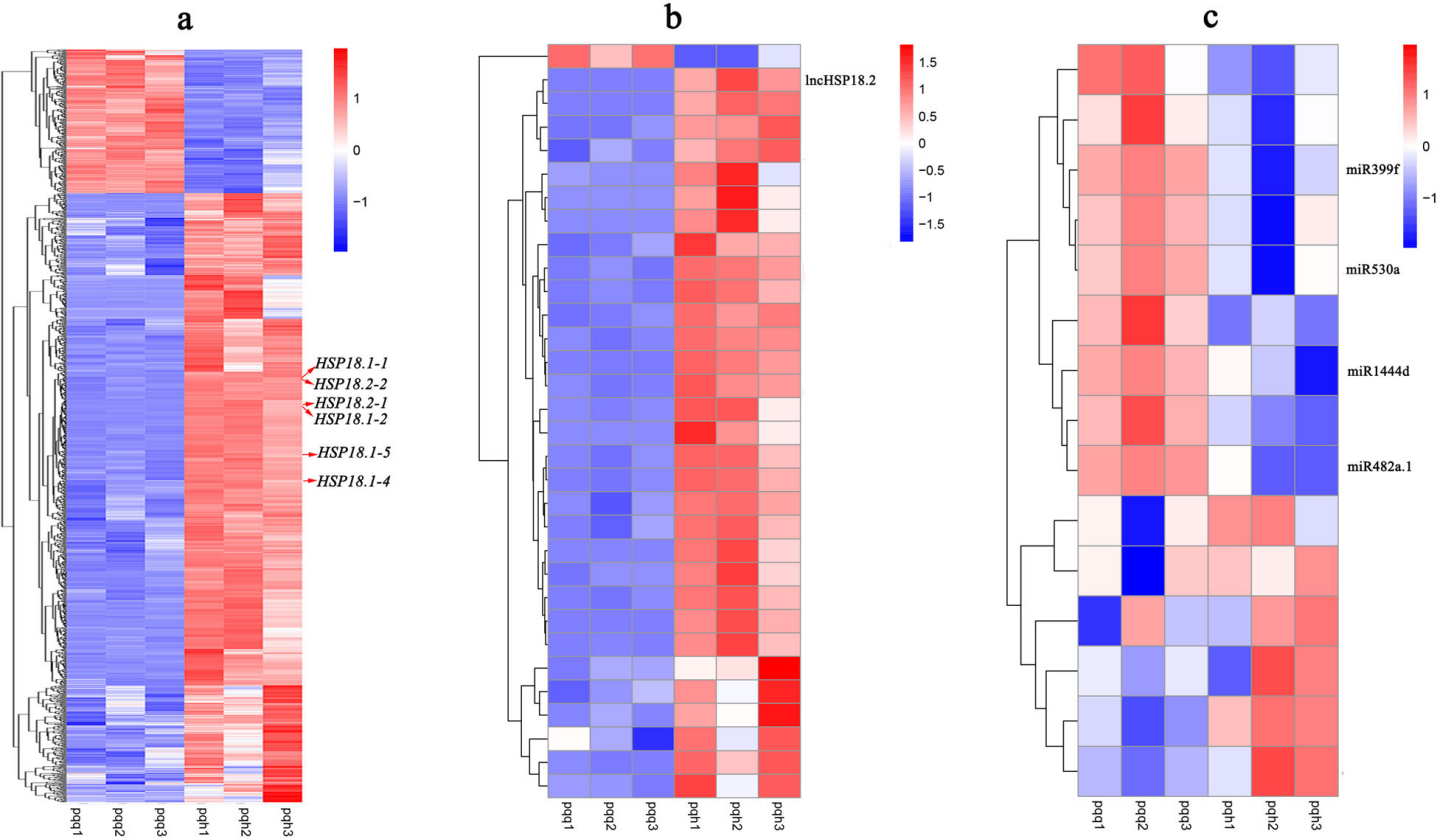
Expression profiles of mRNAs, miRNAs, and lncRNAs in *P. qiongdaoensis*. Hierarchical clustering of all differentially expressed mRNAs (**a**), lncRNAs (**b**) and miRNAs (**c**) in the pqq groups and pqh groups

To investigate the gene expression patterns of *P. qiongdaoensis* seedlings between the heat-stress treatment and control groups, the Fragments Per Kilobase of exon model per Million mapped reads (FPKM) values were used to normalize the reads from RNA-seq to robustly compare relative gene expression levels between the control and experimental groups. A total of 1690 mRNAs were detected as being differentially expressed by the high-throughput sequencing under |log_2_fold-change| ≥ 1 (log_2_FC) and *P*-value < 0.05 (Fig. 2a). Among them, 1296 and 394 were up-regulated and down-regulated in six seedlings, respectively. GO enrichment analysis of significantly differentially expressed mRNAs showed that the up-regulated mRNAs were associated with 913 biological processes, 461 molecular functions, and 221 cellular components, such as unfolded protein binding and heat shock protein binding (Supplementary Fig. S6a). The down-regulated mRNAs were associated with 429 biological processes, 312 molecular functions and 106 cellular components, such as transferase activity and fructose metabolic process (Supplementary Fig. S6b). KEGG pathway enrichment analysis applied on the differentially expressed mRNAs showed that the up-regulated mRNAs were associated with 71 pathways, such as protein processing in endoplasmic reticulum (Supplementary Fig. S7a), while the down-regulated mRNAs were associated with 62 pathways, such as brassinosteroid biosynthesis and phenylpropanoid biosynthesis (Supplementary Fig. S7b).

Importantly, 26 differentially expressed lncRNAs were obtained, consisting of 25 up-regulated lncRNAs and one down-regulated lncRNA (Fig. 2b). We identified 595 candidate target genes for the differentially expressed lncRNAs target genes, of which 12 target genes were differentially expressed. Only one case of down-regulated differential expression of lncRNA was found, so the up-regulated and down-regulated lncRNA target genes were combined for the enrichment analysis. The GO term analysis showed that most of the genes participate in biological regulation: there were 92 for biological regulation, 36 for cellular component, and 19 for molecular function such as NADP binding and phosphogluconate dehydrogenase (decarboxylating) activity (Supplementary Fig. S6c). According to the KEGG analysis, four pathways were enriched such as protein processing in endoplasmic reticulum (Supplementary Fig. S7c).

A total 15 differentially expressed miRNAs were acquired, of which six miRNAs (miR166a, miR403c-5p, miR319e, miR1447, miR156g, miR167f-3p) were up-regulated and nine miRNAs (miR403a, miR530a, miR394a-5p, miR160e-5p, miR6472, miR1444b, miR482a.1, miR1444d, miR399f) were down-regulated when compared with the pqq groups by small RNA-seq (Fig. 2c). The top up-regulated miRNAs included miR166a (1.7-FC), miR319e (1.5-FC), and miR403c-5p (1.3-FC), while the top down-regulated miRNAs included miR399f (− 2.5-FC), miR1444d (− 1.0-FC), miR482a.1 (− 1.6-FC), miR1444b (− 1.6-FC), and miR6472 (− 1.6-FC). However, we did not detect significant differential expression of novel miRNAs according to our screening criteria (*P*-value < 0.05, |log_2_FC| > 1). We identified 563 candidate target genes for the differentially expressed miRNA-target genes, of which 10 target genes were differentially expressed, and 39 miRNA–target gene pairs had significant co-expression levels (Supplementary Fig. S8). GO enrichment analysis of miRNA-target genes showed that the target genes of up-regulated miRNAs were associated with eight biological processes, five molecular functions and nine cellular components, such as mitochondrial fusion and organelle fusion (Supplementary Fig. S6d). The target genes of down-regulated miRNAs were associated with seven biological processes, seven molecular functions and eight cellular components, such as nucleic acid binding (Supplementary Fig. S6e). KEGG pathway enrichment analysis applied on the differentially expressed miRNAs revealed that the up-regulated mRNAs targeted by miRNAs were associated with 18 pathways, such as amino sugar and nucleotide sugar metabolism, plant hormone signal transduction, and spliceosome (Supplementary Fig. S7d), while the down-regulated mRNAs were associated with 19 pathways, such as amino sugar and nucleotide sugar metabolism, plant hormone signal transduction, spliceosome, and RNA transport (Supplementary Fig. S7e).

### LncRNA regulated *HSP18.2* in response to heat stress in *P. qiongdaoensis*

**Fig. 3:**
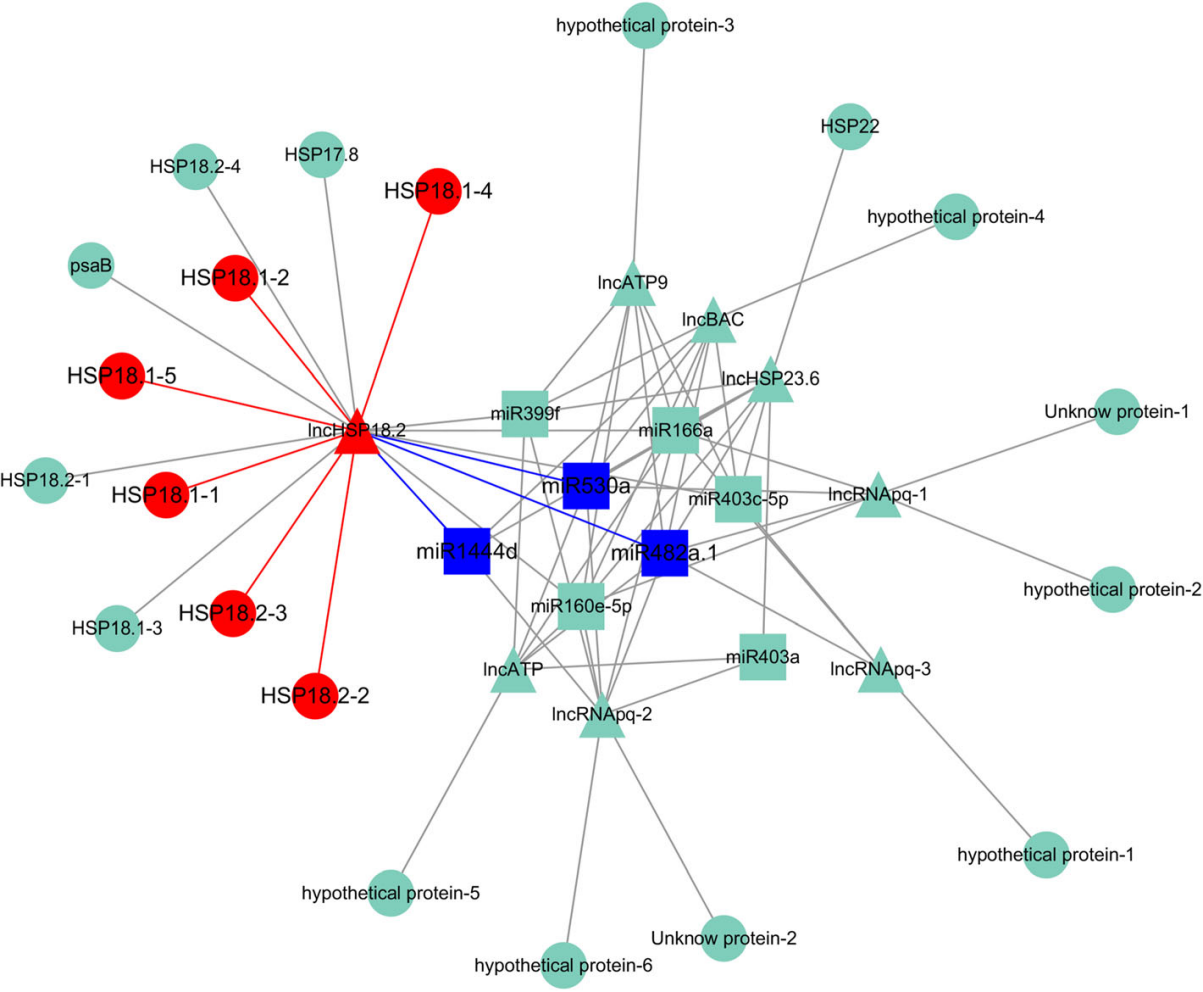
Key regulation networks of miRNAs-lncRNA-mRNA in *P. qiongdaoensis*. The square is the miRNA, the triangle is the lncRNA, and the circle is the mRNA. The red indicates up-regulated, and the blue indicates down-regulated

**Fig. 4:**
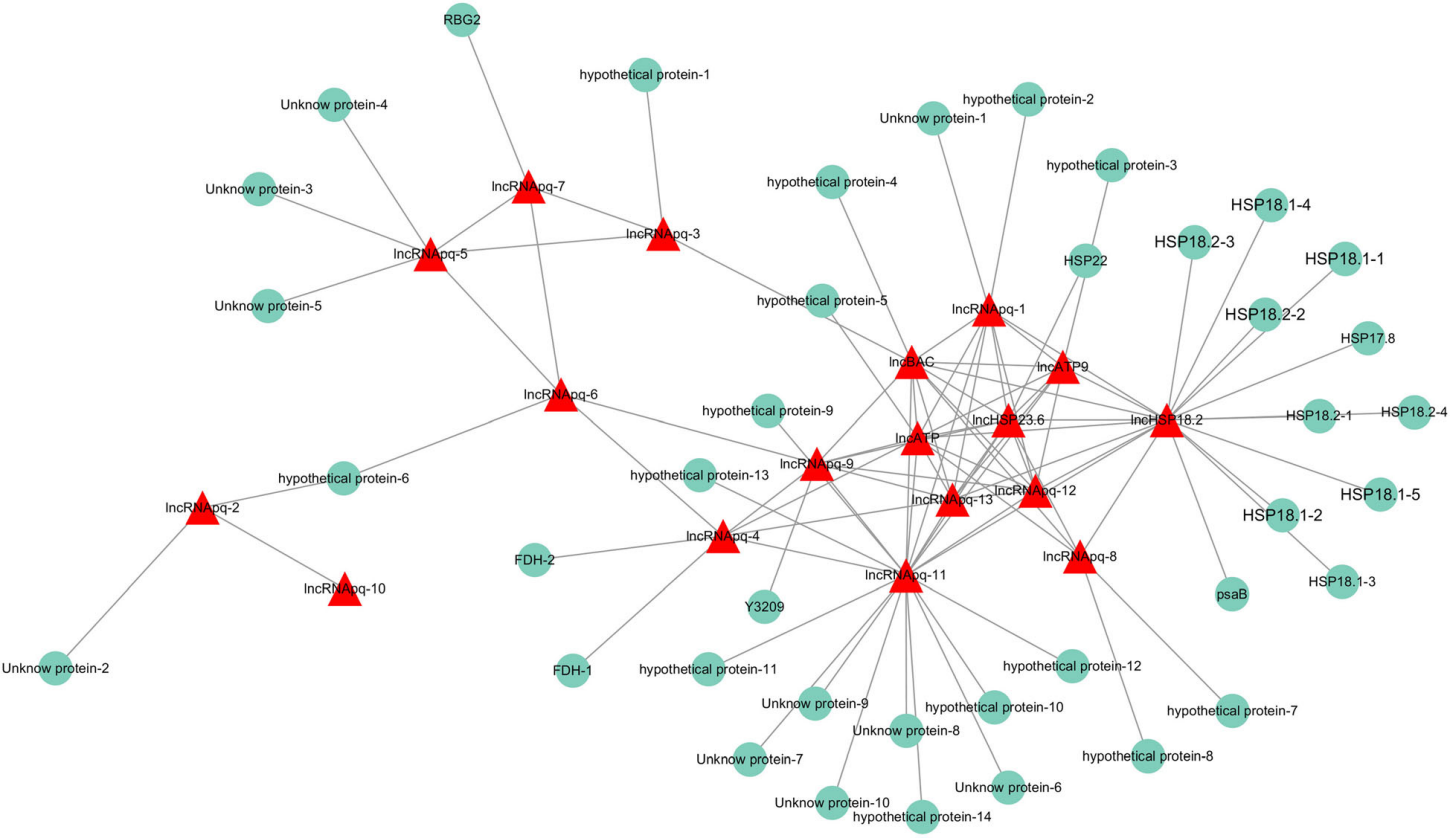
Networks of lncRNAs and mRNAs in *P. qiongdaoensis*. The red triangle is the lncRNA, and the green circle is the mRNA

The miRNA–lncRNA–mRNA co-expression network was constructed using combined miRNA–lncRNA related pairs and the lncRNA potential target genes (Figs. 3 and 4). A total of eight miRNAs, eight lncRNAs, and 20 mRNAs formed this co-expression network (Fig. 3), for which six miRNAs and one mRNA were down-regulated, yet two miRNAs, eight lncRNAs, and 19 mRNAs were found up-regulated. This indicated that miRNAs may negatively regulate the lncRNAs and mRNAs in the tree seedlings’ response to high-temperature stress. Annotating the genes in the network revealed most of them to be heat shock protein genes (Table 2). Analysis of GO terms for mRNA indicated that most of the genes in the co-regulatory pair participate in protein formation. But the KEGG pathway analysis only found a single significant enrichment pathway associated with protein synthesis. In the general miRNA–lncRNA–mRNA network, we found 71 regulatory pathway in network that may be related to high-temperature stress resistance in *P. qiongdaoensis*. Of these 71 regulatory pathway, 42 were significantly correlated with high-temperature stress by enrichment analysis and KEGG pathway analysis of mRNA; which included six up-regulated genes (*HSP18.1–1*, *HSP18.1–2*, *HSP18.1–4*, *HSP18.1–5*, *HSP18.2–2*, *HSP18.2–3*), one up-regulated lncRNA (lncHSP18.2), two up-regulated miRNAs (miR166a, miR403c-5p), and five down-regulated miRNAs (miR1444d, miR160e-5p, miR399f, miR482a.1, miR530a).

**Table 2:** Differentially expressed genes in miRNA–lncRNA–mRNA network

Gene ID	Annotation	FPKM (pqq)	FPKM (pqh)	log_2_FC	padj	Up/ Down regulation
i1_HQ_Pq_c8712/f2p23/1723	*HSP18.1–1*	1.58	863.66	10.48	8.90E-06	Up
i0_HQ_Pq_c14828/f2p1/773	*HSP18.1–2*	0.073	26.77	9.73	5.75E-06	Up
i1_LQ_Pq_c15779/f1p21/1067	*HSP18.1–4*	3.36	4862.22	11.9	8.86E-03	Up
i0_LQ_Pq_c5978/f1p0/868	*HSP18.1–5*	0	5.54	Inf	3.22E-05	Up
i2_LQ_Pq_c36814/f1p62/2615	*PSAB*	0	0.18	Inf	7.43E-03	Up
i1_LQ_Pq_c3954/f1p26/1521	*HSP18.2–1*	0.08	37.79	10.28	6.66E-05	Up
i0_LQ_Pq_c3526/f1p0/1014	*HSP18.2–2*	0.18	116.36	10.72	6.66E-08	Up
i0_LQ_Pq_c3196/f1p0/809	*HSP17.8*	0	3.69	Inf	1.56E-03	Up
i4_LQ_Pq_c2475/f1p0/4683	hypothetical protein	0.003	0.11	6.23	1.50E-02	Up
i4_LQ_Pq_c3767/f1p2/4265	hypothetical protein	0.017	1.28	7.614	2.94E-04	Up
i1_LQ_Pq_c12814/f1p0/1512	Unknown gene	0.017	0.35	5.344	3.47E-02	Up

We used RNAfold to predict the secondary structure of the key lncHSP18.2 and *HSP18.2*, which provide their optimal secondary structure in dot-bracket notation (Supplementary Fig. S9a; Supplementary Fig. S10a), their minimum free energy secondary structure (Supplementary Fig. S9b; Supplementary Fig. S10b), as well as their minimum free energy (MFE), which was − 210.00 kcal/mol and − 278.10 kcal/mol, respectively. The centroid secondary structure of lncHSP18.2 and *HSP18.2* (Supplementary Fig. S9c; Supplementary Fig. S10c), as well as the free energy of the thermodynamic ensemble, which was − 223.18 kcal/mol and − 295.85 kcal/mol, respectively. The minimum free energy of the centroid secondary structure for lncHSP18.2 and *HSP18.2* as indicated by the dot-bracket notation was − 148.31 kcal / mol and − 256.69 kcal/mol, respectively, for which the corresponding frequency of the MFE structure in the ensemble was 5.17 × 10^− 10^ and 3.09 × 10^− 13^, with an ensemble diversity of 226.27 and 189.17. The mountain map of lncHSP18.2 and *HSP18.2* conveys the number of bases corresponding to the MFE structure, the thermodynamic ensemble of RNA structures, and the centroid structure, as well as the correspond loops and helices (Supplementary Fig. S9d; Supplementary Fig. S10d). Together, these results showed that the secondary structures of *HSP18.2* and lncHSP18.2 differ significantly, with former’s stability higher than the latter’s.

### Validation of miRNA, lncRNA and gene expression by qRT-PCR

**Fig. 5:**
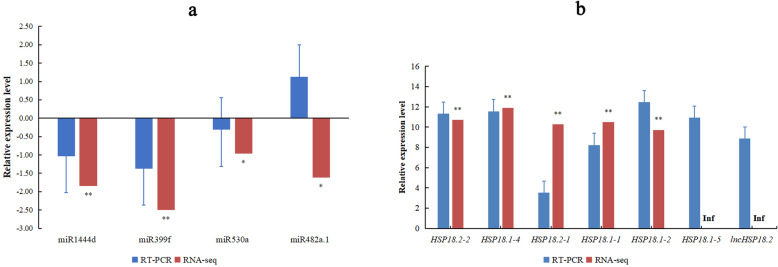
Differential expression analysis of miRNAs, genes and lncRNA in miRNA–lncRNA–mRNA network. The expression results of miRNAs (**a**), genes and lncRNA (**b**) in RT-PCR and RNA-seq. ** mean significant difference between control and heat stress at *P* ≤ 0.01. Inf mean the expression level of genes is zero in the control

Compared with the expression of the control miRNAs, lncRNA and genes, the expression patterns of the four miRNAs, one lncRNA and six genes after the heat-stress treatment showed only miR482a.1 was different with RNA-seq, and the expression patterns of other miRNAs, lncRNA and genes showed similar trends between the high-throughput sequencing and qRT-PCR (Fig. 5a). We noticed that the FC in their expression calculated by sequencing did not exactly match the expression values as detected by qRT-PCR, but the expression profiles were basically consistent for all six genes tested (Fig. 5b). These analyses confirmed the reliability of the gene expression values generated from sequencing results.

## Discussion

### Full-length sequences identified by SMRT sequencing in *P. qiongdaoensis* transcriptome improved tree physiology studies of heat stress

In recent years, high-throughput sequencing technology has greatly facilitated the study of transcriptomes. With advances in sequencing technology, the emergence of SMRT sequencing has greatly enabled the de novo assembly of transcriptomes in higher organisms [20, 21]. In our study, we first used high-throughput sequencing to obtain high-quality short sequence reads of the *P. qiongdaoensis* transcriptome, for which its full-length sequence reads were then obtained based on SMRT technology. Then we explored transcriptomic changes in the ephemeral tree *P. qiongdaoensis* in response to a continuous heat-stress treatment, by combining SMRT sequencing with Illumina sequencing, and demonstrated that this approach provides high-quality results and a more complete assembly [20–22]. Specifically, we obtained 88,161 complete transcripts for *P. qiongdaorensis* by using short reads to correct the long reads of SMRT, which greatly improved the accuracy and depth of the study overall. This paper thus provides accurate transcriptome data for use in subsequent studies. Further, it is the first study to perform SMRT sequencing of the full-length transcriptome of the *P. qiongdaoensis* tree. We anticipate the obtained transcriptome may spur and assist further exploration of this tree’s genetics and physiology.

### miRNAs regulated lncRNAs and mRNAs in response to heat stress in *P. qiongdaoensis*

Studying the molecular mechanism underpinning the responses of plants under high-temperature stress is the key to breeding heat-resistant varieties. Especially involved in this are key regulatory components such as miRNA. In this study, miRNAs were identified in six samples using sRNA sequencing: several of them were known miRNAs supported by previous studies, but 21 were novel miRNAs. In particular, Xin et al. reported a series of known miRNAs to be involved in heat-stress responses, namely miR156, miR160, and miR166 [23]. Later work found the miR160 and miR166 families highly expressed in rice and wheat plants under heat stress [24, 25]. In our study, both miR156 and miR166 were significantly up-regulated in the pqh compared with the pqq group, which is consistent with previous studies, but the miR160 was significantly down-regulated in *P. qiongdaoensis*. Li et al. reported that miR482, miR1447, and miR1444 were all involved in the heat-stress response of *Populus* trees [26]. In our study, miR482, miR1447, and miR1444 were also differentially expressed, and their target genes were involved in protein synthesis, such as the synthesis of heat shock proteins. Therefore, we believe that these miRNAs may be crucial factors in the heat shock reaction process of *P. qiongdaoensis*. In addition, this study provides more details concerning the range of expression patterns of miRNAs involved in this process, in that we several known miRNAs were found differentially expressed under heat stress, such as miR403, miR319, miR1447, miR167, miR399, miR6472, and miR394. Although previous studies have examined miRNAs involved in heat stress, more miRNAs still need to be found in other tree species.

Mounting evidence points to the existence of a broad regulatory interaction between miRNAs and lncRNAs [27]. Our study built a comprehensive network of RNA-mediated interactions, putting together the miRNA–lncRNA, miRNA–mRNA, and lncRNA–mRNA interactions (Fig. 4; Supplementary Fig. S8), by using a computational approach (Fig. 3). In this way, a network of interactions between eight miRNAs, eight lncRNAs, and 20 mRNAs was constructed, showing that miRNAs may function through lncRNA pathways and corroborating the interactions between miRNAs and lncRNAs. Our study identified eight lncRNAs that might be targeted by eight miRNAs, and we found that six miRNAs were negatively correlated with eight lncRNAs; this result agrees with earlier studies that showed miRNAs could target lncRNAs, with widespread regulatory interactions occurring between non-coding RNAs and mRNAs in *Populus* trees [27, 28]. Further, in miRNA–lncRNA–mRNA network, there six miRNAs and one mRNA down-regulated, but two miRNAs, eight lncRNAs, and 19 mRNAs up-regulated. This result suggests that miRNAs negatively regulate lncRNA action on mRNA in the tree’s response to heat stress. The mRNAs in the miRNA–lncRNA–mRNA network were subjected to GO and KEGG enrichment analysis. From the KEGG results, we found that mRNA was significantly enriched in protein processing in the endoplasmic reticulum pathway, with gene annotations confirming that most genes are involved in the synthesis of heat shock proteins. By enriching the analysis results, we screened a key lncRNA and its target gene annotations were indeed heat shock protein synthesis genes. This result strongly implicates lncRNA as being associated with resistance to heat stress in trees. According to the above results, the mutual regulation of miRNA, lncRNA, and mRNA in plants likely plays a vital role in how they resist or tolerate high-temperature stress.

### LncRNA is involved in the regulation of heat-stress response in *P. qiongdaoensis*

Many molecular mechanisms regulate the variation in expression of one or more genes, including *cis*-regulatory elements that regulate gene transcription and genetic variation of trans-acting factors [29, 30]. Non-coding RNAs, especially lncRNAs, may regulate gene expression at various levels in the form of RNA [31]. In our study, 928 putative lncRNAs were identified by transcriptome sequencing and divided into four categories using computational analysis to explore their possible functions. Since the genome-wide sequencing of *P. qiongdaoensis* has not yet been achieved, we used the genome of *P. trichocarpa* as a reference. Previous studies have shown that lncRNA is critically involved in plant development and stress responses [28, 32]. For example, Di et al. found differential expression of lncRNAs in Arabidopsis under heat stress [33]. In *Brassica rapa*, 34 specifically expressed lncRNAs were identified when this plant was under heat stress, with most of the lncRNA target genes belonging to *HSP* genes [34]. In our study, 25 lncRNAs showed a unique expression pattern in response to heat stress, suggesting that some lncRNAs are closely involved in governing the heat-stress responses of *P. qiongdaoensis* trees. To understand the function of lncRNA in heat-stress responses, our study thus provides a useful method for predicting the trans-regulated target gene of lncRNA, which can be then used to identify those processes that lncRNA is involved in and there by infer its potential function [35, 36]. Here, we identified 71 target genes for differentially expressed lncRNAs, by using the counter-regulatory effect of sequence complementarity. Functional annotation of the target gene revealed that most of these target genes are involved in heat stress response. This result further confirmed that lncRNAs likely plays an important regulatory role in *P. qiongdaoensis* under heat stress.

Through the interaction network we found three key miRNAs (miR1444d, miR482a.1, miR530a) that may figure prominently in how trees respond to high-temperature stress; all three were reportedly involved in the stress response of *Populus* [37]. But in our study, we also uncovered significant differential expression of miR1444d, miR482a.1, and miR530a, with miR530a targeting *HSP70*, and miR482a.1 targeting both *sHSP* and *HSP23.6*. In addition, we screened a key lncRNA (lncHSP18.2), and its six target genes (*HSP18.1–1*, *HSP18.1–2*, *HSP18.1–4*, *HSP18.1–5*, *HSP18.2–2*, *HSP18.2–3*). The lncHSP18.2 was aligned to the *P. qiongdaoensis* transcriptome data by sequence alignment, and the target genes were annotated as *HSP18.1* and *HSP18.2*. To verify the accuracy of transcriptome sequencing, the expression levels of three miRNAs, lncHSP18.2 and six genes were cross-checked by qRT-PCR. These qRT-PCR results were consistent with our RNA-seq data regarding the *HSP* expression levels, and this result is also consistent with previous studies [38]. These results suggested that these lncRNAs identified from third-generation sequencing may regulate the *HSP* genes through co-expression. These results also suggested that lncRNA18.2 may cis-regulate the *HSP18.2* co-expression.

## Conclusions

This study is the first to characterize the transcriptome of *P. qiongdaoensis* under heat stress. Many differentially expressed miRNAs, lncRNAs and genes were screened and identified. Functional enrichment analysis indicates that these miRNAs, lncRNAs and genes play an important role in the response to heat stress *P. qiongdaoensis*. According to the interaction analysis of the miRNAs, lncRNAs and genes, the miRNA–lncRNA–mRNA network of this species was constructed. By investigating the miRNA–lncRNA–mRNA network, we revealed that miRNAs may negatively regulate both lncRNAs and mRNAs in tree responses to heat stress, and found that lncHSP18.2 may *cis*-regulate *HSP18.2*.

## Methods

### Plant materials and heat-stress treatment

*P. qiongdaoensis* used in the study is the only *Populus* in tropical China, it was published by Luo and Hong as a new species in 1987 [39], and the voucher specimen was deposited in the Herbarium of Forest Plants, Chinese Academy of Forestry. Its identification of the chloroplast genome was completed in 2016 [17]. In our study, the seeds of *P. qiongdaoensis* were collected from Ba King ridge in Hainan (109°04′ E, 109°27′ N). These seeds were planted in Hainan University and had grown for 5 months (Danzhou; 109°29′ 25“ E, 19°30’ 40” N). Uniformly developed seedlings of *P. qiongdaoensis* were selected for the heat treatment experiment. The plants were placed in an experimental greenhouse at Hainan University and had grown for 1 months, after which six uniformly sized individuals were taken. Specifically, three biological replicate seedlings (pqh1, pqh2, and pqh3) received the heat-stress treatment at 40 °C for 1 h, and another three (pqq1, pqq2, and pqq3) served as the controls. The collected six seedling leaves were immediately frozen in liquid nitrogen. We further stored the leaves at − 80 °C. for follow-up RNA extraction experiments,

### RNA isolation and RNA-seq

Mature non-senescent leaves were collected from the pqq (control) and pqh (heat-stress treatment) groups. Only those RNA samples with OD_260/280_ values of 1.9 to 2.2, OD_260/230_ values ≥2.0, and RNA integrity number (RIN) values > 6.8 were used for the subsequent experiments using the NanoDrop 2000 and the Agilent 2100 Bioanalyzer. Then, mRNA of containing polyA was enriched by using oligo (dT), and the mRNA was broken into short fragments by adding fragmentation buffer. We then use random hexamers to reverse transcribe mRNA into single-stranded cDNA. Add dNTP, DNA polymerase I and buffer to synthesize two-stranded cDNA. The two-stranded cDNA was purified, by using AMPure XP beads, then the end repair, A-tail binding, sequencing linker binding, and select fragment size were performed. Finally, PCR enrichments were performed to construct a final cDNA library. The final cDNA libraries were sequenced on the Illumina HiSeq 2500 platform (Illumina, San Diego, CA, USA).

The small RNA-seq libraries were generated using NEBNext® Multiplex Small RNA Library Prep Set for Illumina® (NEB, Ipswich, MA, USA.) by following the manufacturer’s instructions. After using TruSeq SR Cluster Kit v3-cBot-HS (Illumina) to generate cluster on cBot Cluster Generation System, the small RNA-seq libraries were all sequenced on an Illumina HiSeq 2500 platform.

### PacBio SMRTbell library preparation

To prepare the SMRTbell library, equal amounts of RNA samples from the biological replicates—same seedlings previously used for the RNA-seq libraries—were combined to generate one pools (control and heat stress). First, mRNA containing polyA was enriched by using oligo (dT); Using SMARTer PCR cDNA synthesis kit to synthesize cDNA with mRNA as template. Finally, optimize the optimal conditions and amplify cDNA by PCR to obtain the final library. We performed injury repair, end repair, binding of SMRT dumbbell-type linker and SMRTbell library construction for full-length cDNA [40]. The unbound sequences at both ends of the cDNA were removed. Then, a complete SMRT Bell libraries were constructed by binding primers and DNA polymerase. The sequencing was performed using PacBio Sequel II System at Novogene Bioinformatics Technology Co., Ltd. (Beijing, China). The subreads sequences were obtained by processing the raw sequence data on SMRTlink v6.0 software. CCS was derived from the correction between the subreads. Specifically, using CCS, each sequence was further divided into a full-length sequence and a non-full-length sequence according to whether the sequence contained 5′ primer, 3′ primer, and polyA tail [41]. The full-length sequences were clustered via ICE algorithm to obtain the consensus sequences. Finally, this obtained consensus sequences were then calibrated with non-full-length sequences, yielding high-quality sequence for follow-up analysis.

### Functional annotation and lncRNA identification

To investigate the functions of all the non-redundant transcripts, BLAST v2.2.26 [42], KOBAS v2.0 [43] and HMMER v3.1 [44] software tools were used to search the following public databases: NR [45], Nt [45], KOG [46], COG [47], Swiss-Prot [48], KEGG [49], GO [50], and Pfam [51].

To identify the lncRNAs in the Iso-seq data, we used four analysis methods—namely CPC v0.9 [52], CNCI v2 [53], PLEK v1.2 [54] and PfamScan v1.6 [51]. CPC used to assess the degree and quality of ORFs in transcripts, set the e value to “1e-10”, and use the NCBI eukaryote protein database search sequence to distinguish between coding and non-coding transcripts. CNCI used the default parameters. The setting parameters for searching Pfam was -E 0.001 --domE 0.001. PLEK used the default parameter of -minlength 200 to evaluate the coding potential of transcripts that lack genome sequences and annotations, and delete transcripts with a predicted length of < 200 bp.

Then transcripts without coding potential were selected as our candidate lncRNAs. Due to the lack of a complete genome sequence of *P. qiongdaoensis*, lncRNA sequences were aligned to *P. trichocarpa* genome (3.0) and were classified into four categories, including sense lncRNAs, lincRNAs, antisense lncRNAs and sense intronic lncRNAs [55–61].

### Data analysis

Gene expression levels were identified by RSEM v1.3.0 [61] for six samples. LoRDEC software was used to map RNA-seq data to Iso-seq data to obtain the corrected consensus sequences. To determine the gene expression levels in a given plant’s response to heat stress, the corrected consensus sequence was de-redundified using CD-HIT (−c 0.95 -T 6 -G 0 - aL 0.00 -aS 0.99), and the ensuing full-length transcripts used as a *ref* for that particular gene; next, align the clean reads obtained by Illumina sequencing to *ref*, and the read-count of all genes was obtained. Genes with FPKM values > 0.3 in samples from two groups (heat stress and control) were selected for further analysis [62, 63]. The DESeq R software package (1.10.1) was used for the expression analysis. In order to controll the false discovery rate, Hochberg and Benjamini were used, and the DEGs were designated as those having FC of |log_2_FC| > 1 and *P*-value < 0.05 [64].

A small RNA-seq library was constructed with the Small RNA Sample Pre Kit, and then they were sequenced on HiSeq 2500 instrument. Clean reads were obtained by removing the reads of quality value (sQ) ≤ 20, ≥ 10% unidentified nucleotides, containing poly-N and poly A /T / G /C, with 5’adaptor contaminants, without 3’adaptor and low quality reads from raw reads. After the Q20, Q30 and GC contents were calculated, the sequences with a length range of 18–30 bp from the clean readings were selected to do the downstream analysis. The small RNA tags were mapped to *P. trichocarpa* genome by Bowtie-0.12.9 (Set the parameter to “-v 0 -k 1”) [65] to search known miRNA. Using the miRBase20.0 as a reference, modified software mirdeep2_0_0_5, set the parameter to “quantifier.pl -p -m -r -y -g 0 -T 10”, and potential miRNAs were obtained using srna-tools-cli. Novel miRNAs were identified using miREvo (set the parameter to “-i -r -M -m -k -p 10 -g 50000”) [66] and mirdeep2 software. The TPM (transcript per million) value was used to estimate the expression level of miRNA [67]. The DEGseq R package (1.8.3) was used for the differential expression analysis with *P*-value < 0.05 as the threshold.

### Predicting the potential target genes and enrichment analysis

Target gene prediction for the lncRNAs was into *cis* and trans prediction. To predict lncRNA target genes, BLAST was used to search for transcriptome libraries of our third-generation sets of sequences, set the e-value to “1e-5” and identity = 80%. Next, potential target genes (*r* > 0.8 or *r* < − 0.8) were screened according to the expression correlation coefficients between lncRNAs and mRNAs [35, 36]. Using the psRNATarget to predict (http://plantgrn.noble.org/ psRNATarget/) the target genes of miRNAs with an expectation ≤3 [68]. We predicted the secondary structure of lncRNA using a loop-based energy model in the RNAfold Web (http://rna.tbi.univie.ac.at/).

For enrichment analysis, we mapped all DEGs to the terms of GO database, and calculated the number of genes in each term. Finally, significant enrichment items in the DEG were identified using GOseq software. Their corresponding pathways were obtained by mapping DEGs to the KEGG pathway database.

### RT-PCR validation

The cDNAs were synthesized by reverse transcription of total RNA from six *P. qiongdaoensis* seedlings, pqq and pqh, used in the heat-stress experiment. Primer Premier v5 software was used to design tailored primers for the target genes (Table 3). Six DEGs and one lncRNA in *P. qiongdaoensis* under heat stress were chosen. For the latter, using Green® Premix Ex Taq™ II (Tli RNaseH Plus; Takara, Beijing, China) for qRT-PCR analysis following the manufacturer’s recommendations. The amplification was performed under the conditions of denaturation at 95 °C for 30 s and 40 cycles. The *β-actin* gene served as an internal control for normalization. All six samples were performed with three technical replicates. For the four miRNAs, the qRT-PCR analysis was conducted with a miRNA RT/qPCR Detection Kit (Aidlab, Beijing, China), following the manufacturer’s recommendations, with the following reaction conditions: denaturation at 94 °C for 20 s and 40 cycles of amplification. The *18 s rRNA* served as an internal control for normalization, as described by Song et al. [34].

**Table 3:** Oligonucleotide primers used in real-time PCR assays in this study

Gene	Primer pairs
miR399f	F:GTGCCAAAGGAGAATTGCCCTGAA
miR530a	F:GTGCATTTGCACCTGCACCTTAAA
miR482a.1	F:GGCCTACTCCTCCCATTCCAAAA
miR1444d	F:GCGAACGTTGACCGAATGTGAAAA
*18 s rRNA*	F:CGGCTACCACATCCAAGGAA
	R:GCTGGAATTACCGCGGCT
*β-actin*	F:AGTGCTTCTAAGTTCCGACAG
	R:GGAGGACCATTACAGTTACGC
*HSP18.1–1*	F:TAAATACTCGCTCATTCCTCA
	R:AAGACATCGGTAGATTCACCA
*HSP18.1–2*	F:GGTCTTGACAGTGACTGCCTAA
	R:ACTCCCTACAACACTCCACAA
*HSP18.1–4*	F:CTCAGCTAGCTTTCACGTCGC
	R:CCAGTCTATGCGTGTGCTGGC
*HSP18.1–5*	F:CTTGAGGAGGTTCAGATTGCC
	R:ATCCCATTCCGCGAGTCTTTC
*HSP18.2–2*	F:TGATTCTTGTCCTTCCCTTTC
	R:ATGTTTCTTTGGCGACCTCTG
*HSP18.2–3*	F:GGTCTTGACAGTGACTGTGCCT
	R:TCATTGAAATGCGCATCTTCTATC
lncHSP18.2	F:GTCGCCTAACACTTGCTTT
	R:CATCACTTTATCCCATTCC

## Supplementary information

**Additional file 1 Figure S1.** Annotation result statistics for seven queried databases. **Figure S2.** Length distribution of all sRNA sequences in the six samples. **Figure S3.** First base type of the known miRNA of six samples of 18–30 nt in length. pqh1 (a), pqh2 (b), pqh3 (c), pqq1 (d), pqq2 (e), and pqq3 (f). **Figure S4.** First base type of the novel miRNA of six samples of 18–30 nt in length. pqh1 (a), pqh2 (b), pqh3 (c), pqq1 (d), pqq2 (e), and pqq3 (f). **Figure S5.** Predicted total number of lncRNAs. **Figure S6.** GO analysis of the biological functions of mRNAs, lncRNAs, and miRNAs. GO terms of the up- (a) and down-regulated mRNAs (b), the lncRNAs (c), and the up- (d) and down-regulated miRNAs (e). **Figure S7.** KEGG pathway enrichment analysis of mRNAs, lncRNAs, and miRNAs. KEGG pathway enrichment analysis of the up- (a) and down-regulated mRNAs (b), the lncRNAs(c), and the up- (d) and down-regulated miRNAs (e). **Figure S8.** Networks of miRNAs and mRNAs in *P. qiongdaoensis*. Thirty-nine mRNAs were predicted as potential target genes of nine miRNAs, in the psRNATarget with an expectation ≤3. **Figure S9.** Prediction of lncHSP18.2 secondary structure. The optimal secondary structure in dot-bracket notation (a), minimum free energy secondary structure (b), centroid secondary structure (c), and mountain plot representation of the MFE structure, thermodynamic ensemble of RNA structure, and the centroid structure (d). Base colors in the b and c diagrams represent base-pair probabilities. A mountain plot represents a secondary structure in a plot of height versus position, where the height m (k) is given by the number of bases at position k. The loops correspond to plateaus (hairpin loops are peaks), the helices to slopes. **Figure S10.** Prediction of mRNA (*HSP18.2*) secondary structure. The optimal secondary structure in dot-bracket notation (a), minimum free energy secondary structure (b), centroid secondary structure (c), and mountain plot representation of the MFE structure, thermodynamic ensemble of RNA structure, and the centroid structure (d). Base colors in the b and c diagrams represent base-pair probabilities. A mountain plot represents a secondary structure in a plot of height versus position, where the height m (k) is given by the number of bases at position k. The loops correspond to plateaus (hairpin loops are peaks), the helices to slopes.

**Additional file 2 Table S1.** Top 10 plant species compared according to the NR database. **Table S2.** Read count of known miRNA-mature leaves. **Table S3.** Base type of each position of the known miRNA. **Table S4.** Read count of novel miRNA-mature leaves. **Table S5.** Base type of each position of the novel miRNA. **Table S6.** Mapping of Illumina sequencing reads and reference reads. **Table S7.** Classification of lncRNA predicted under heat stress in *P. qiongdaoensis*.

## Data Availability

The raw sequence data reported in this paper have been deposited in the Genome Sequence Archive [69] in BIG Data Center [70], Beijing Institute of Genomics (BIG), Chinese Academy of Sciences, under accession numbers CRA002159, CRA002160 and CRA002185 that are publicly accessible at https://bigd.big.ac.cn/gsa. The collection of seeds complies with national guidelines. The whole genome of *P. trichocarpa* is used as a reference for lncRNA classification (http://www.phytozome.net/poplar). GO database (http://www.geneontology.org/). KEGG pathway database (http://www.genome.jp/kegg/pathway.html).

## Abbreviations

miRNAs: MicroRNAs
lncRNAs: Long non-coding RNAs
lincRNAs: Long intergenic non-coding RNAs
FC: Fold-change
HSPs: Heat shock proteins
sHSP: Small heat shock protein
DEGs: Differentially expressed genes
SMRT: Single-molecule real-time
small RNA-seq: Small RNA sequencing
RNA-seq: RNA sequencing
CCS: Circular consensus sequence
FLNC: Full-length non-chimeric
ICE: Iterative isoform-clustering
NR: Non-Redundant Protein Database
Nt: Nucleotide Sequences
GO: Gene Ontology
COG: Cluster of Orthologous Groups of proteins
KOG: Eukaryotic Orthologous Groups
Pfam: Protein family
KEGG: Kyoto Encyclopedia of Genes and Genomes
FPKM: Fragments Per Kilobase of exon model per Million mapped reads
MFE: Minimum free energy
RIN: RNA integrity number
*ref*: Reference sequence
TPM: traNscript per million
