# Supplementary material for: Third-generation sequencing found LncRNA associated with heat shock protein response to heat stress in Populus qiongdaoensis seedlings
Source: BMC Genomics. 2020 Aug 24;21:572. doi: 10.1186/s12864-020-06979-z (PMC7444055; doi:10.1186/s12864-020-06979-z)
Supplement: Supplementary file 1 — Additional file 1 Figure S1. Annotation result statistics for seven queried databases. Figure S2. Length distribution of all sRNA sequences in the six samples. Figure S3. First base type of the known miRNA of six samples of 18–30 nt in length. pqh1 (a), pqh2 (b), pqh3 (c), pqq1 (d), pqq2 (e), and pqq3 (f). Figure S4. First base type of the novel miRNA of six samples of 18–30 nt in length. pqh1 (a), pqh2 (b), pqh3 (c), pqq1 (d), pqq2 (e), and pqq3 (f). Figure S5. Predicted total number of lncRNAs. Figure S6. GO analysis of the biological functions of mRNAs, lncRNAs, and miRNAs. GO terms of the up- (a) and down-regulated mRNAs (b), the lncRNAs (c), and the up- (d) and down-regulated miRNAs (e). Figure S7. KEGG pathway enrichment analysis of mRNAs, lncRNAs, and miRNAs. KEGG pathway enrichment analysis of the up- (a) and down-regulated mRNAs (b), the lncRNAs(c), and the up- (d) and down-regulated miRNAs (e). Figure S8. Networks of miRNAs and mRNAs in P. qiongdaoensis. Thirty-nine mRNAs were predicted as potential target genes of nine miRNAs, in the psRNATarget with an expectation ≤3. Figure S9. Prediction of lncHSP18.2 secondary structure. The optimal secondary structure in dot-bracket notation (a), minimum free energy secondary structure (b), centroid secondary structure (c), and mountain plot representation of the MFE structure, thermodynamic ensemble of RNA structure, and the centroid structure (d). Base colors in the b and c diagrams represent base-pair probabilities. A mountain plot represents a secondary structure in a plot of height versus position, where the height m (k) is given by the number of bases at position k. The loops correspond to plateaus (hairpin loops are peaks), the helices to slopes. Figure S10. Prediction of mRNA (HSP18.2) secondary structure. The optimal secondary structure in dot-bracket notation (a), minimum free energy secondary structure (b), centroid secondary structure (c), and mountain plot representation of the MFE structure, thermodynam [file 12864_2020_6979_MOESM1_ESM.pdf]

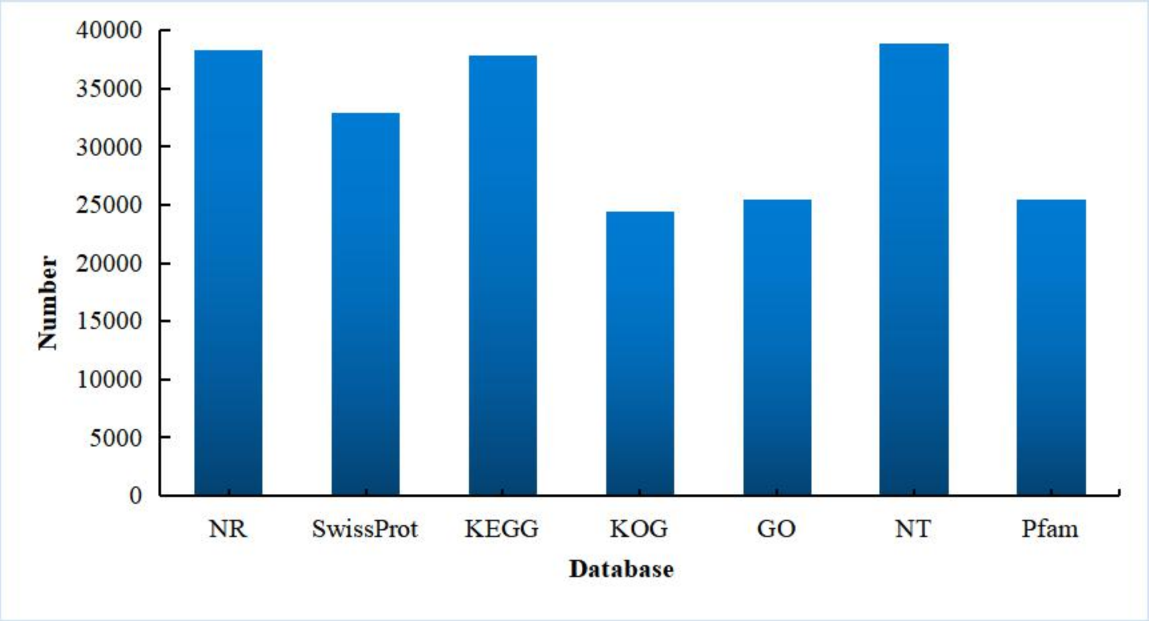

Supplementary Fig. S1 Annotation result statistics for seven queried databases.

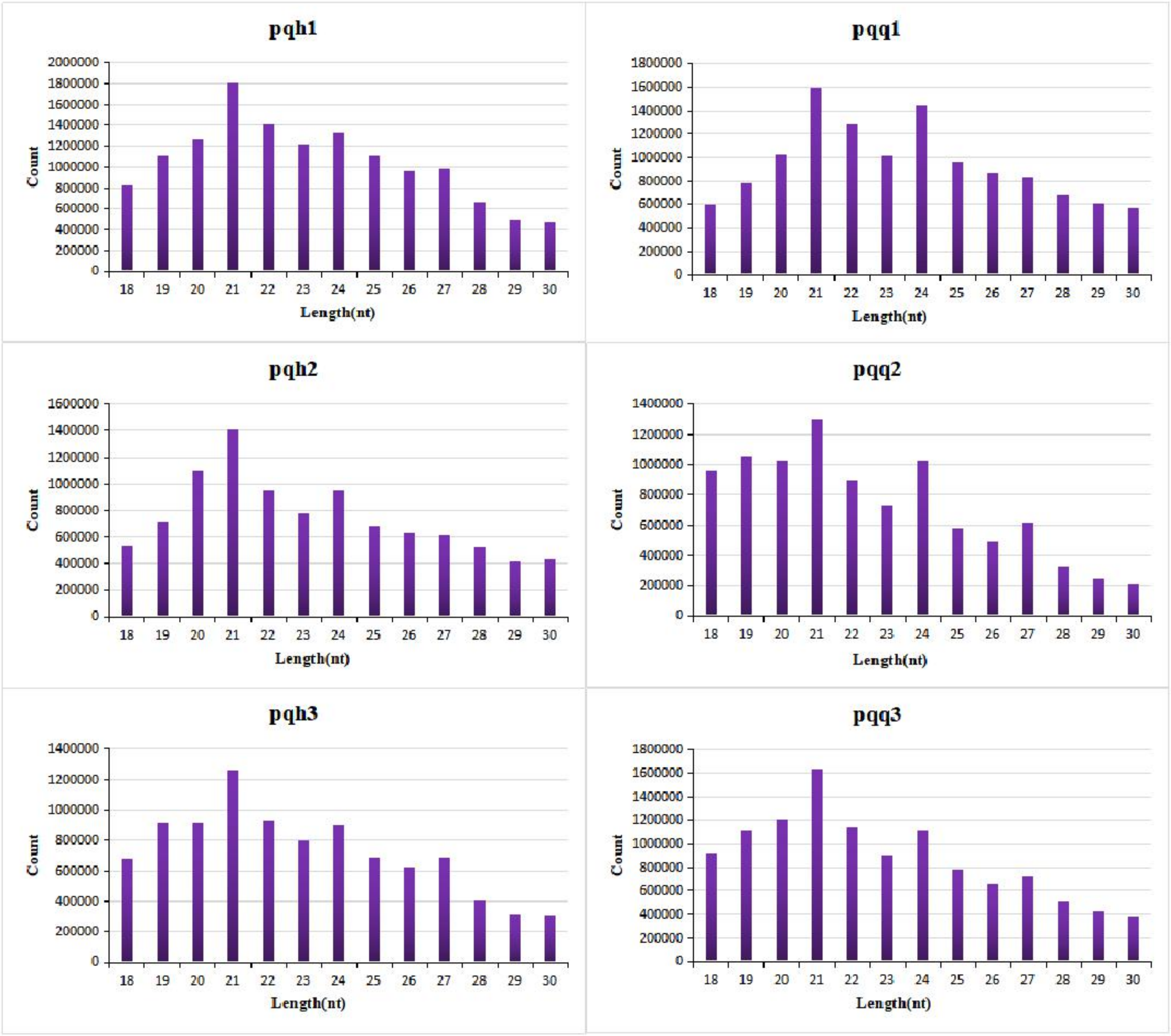

Supplementary Fig. S2 Length distribution of all sRNA sequences in the six samples.

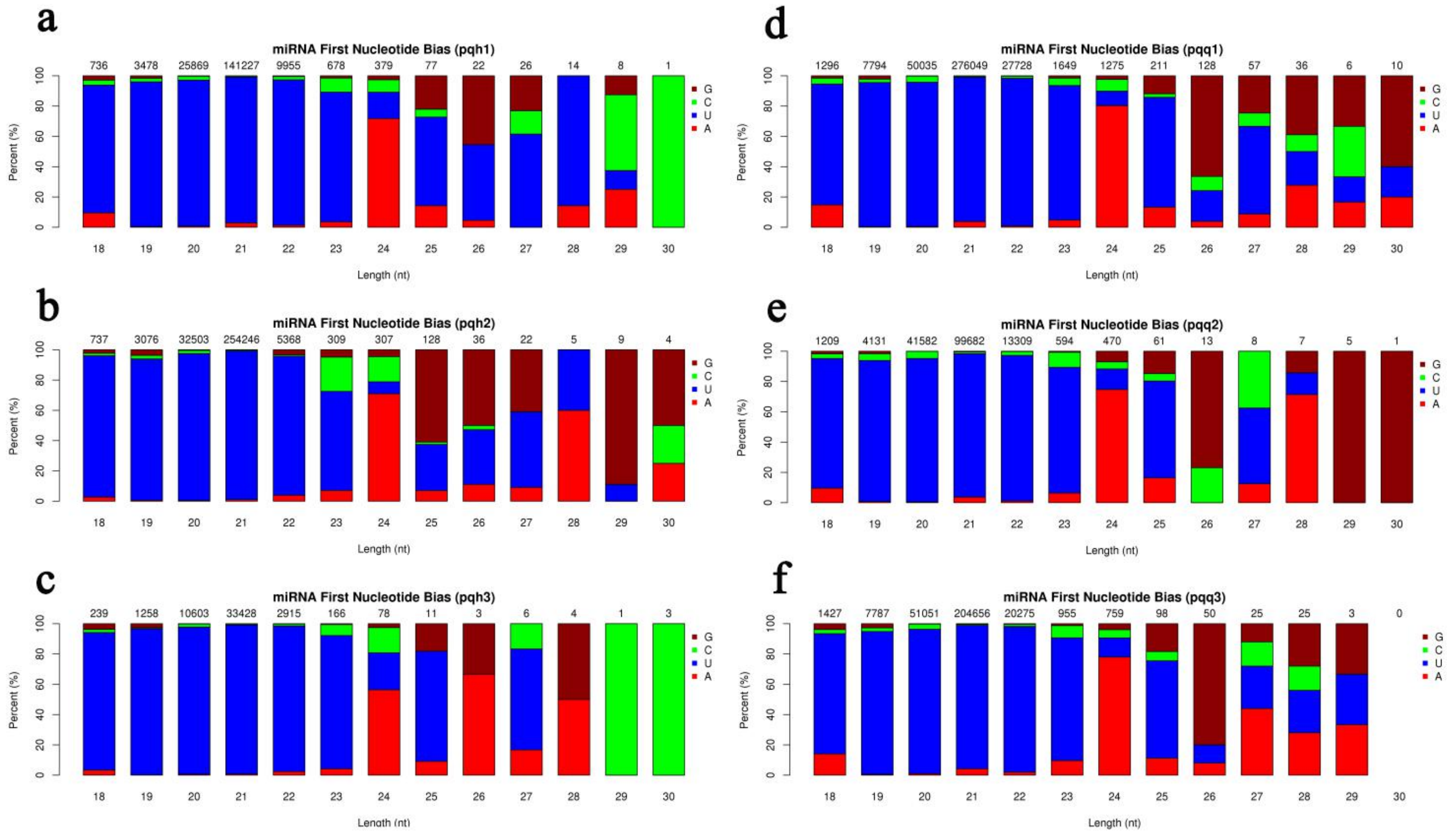

Supplementary Fig. S3 First base type of the known miRNA of six samples of 18–30 nt in length. pqh1 (a), pqh2 (b), pqh3 (c), pqq1 (d), pqq2 (e), and pqq3 (f).

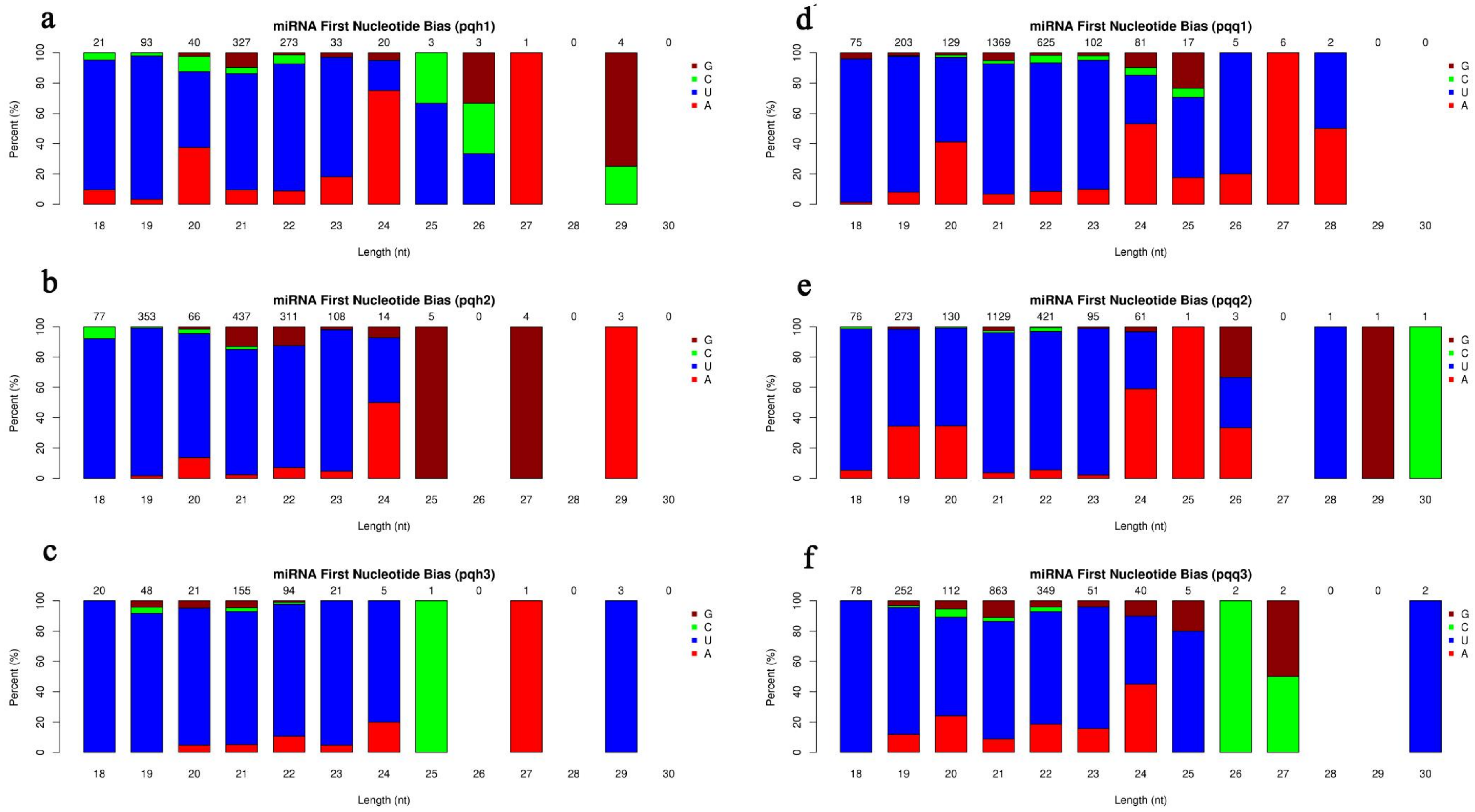

Supplementary Fig. S4 First base type of the novel miRNA of six samples of 18–30 nt in length. pqh1 (a), pqh2 (b), pqh3 (c), pqq1 (d), pqq2 (e), and pqq3 (f).

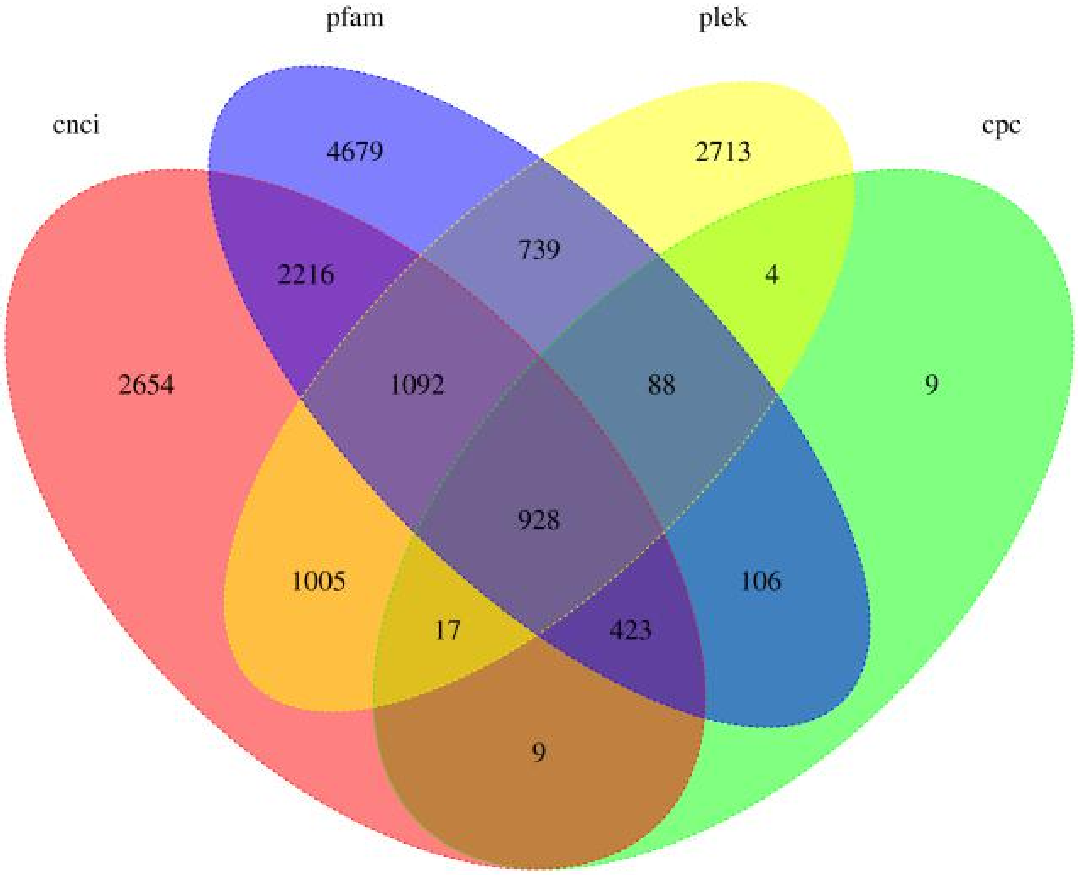

Supplementary Fig. S5 Predicted total number of lncRNAs.

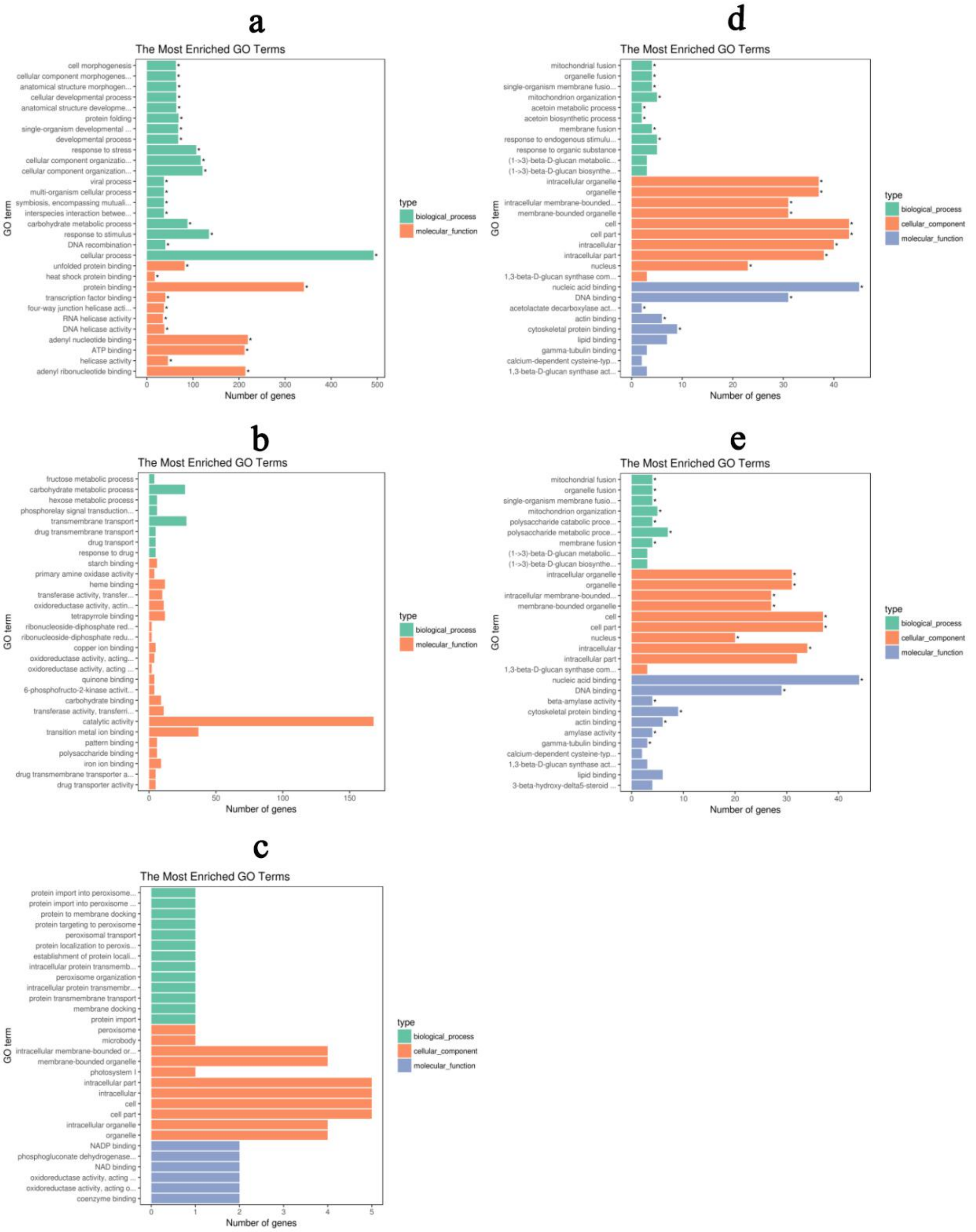

Supplementary Fig. S6 GO analysis of the biological functions of mRNAs, lncRNAs, and miRNAs. GO terms of the up- (a) and down-regulated mRNAs (b), the lncRNAs (c), and the up- (d) and down-regulated miRNAs (e).

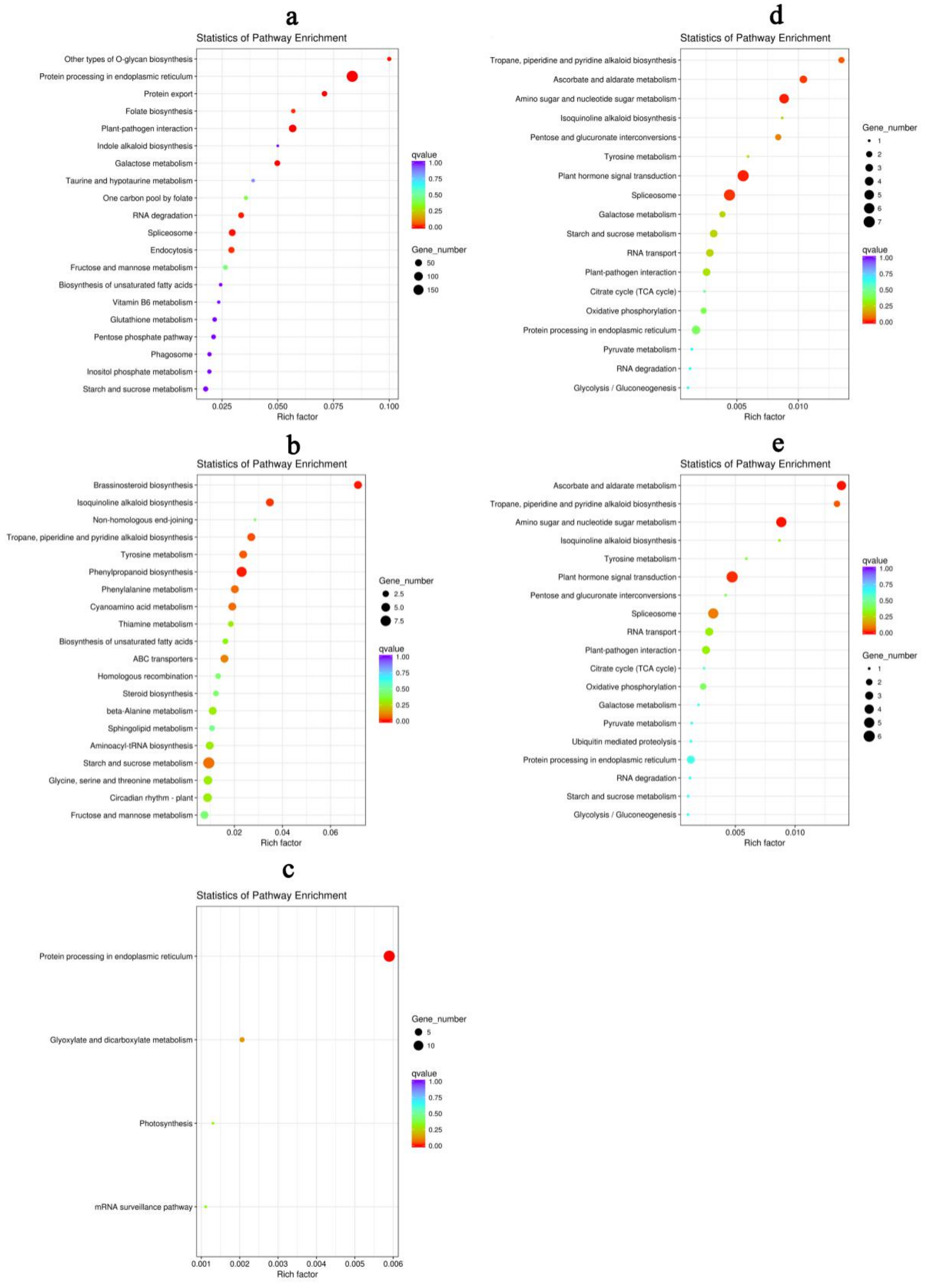

Supplementary Fig. S7 KEGG pathway enrichment analysis of mRNAs, lncRNAs, and miRNAs. KEGG pathway enrichment analysis of the up- (a) and down-regulated mRNAs (b), the lncRNAs(c), and the up- (d) and down-regulated miRNAs (e).

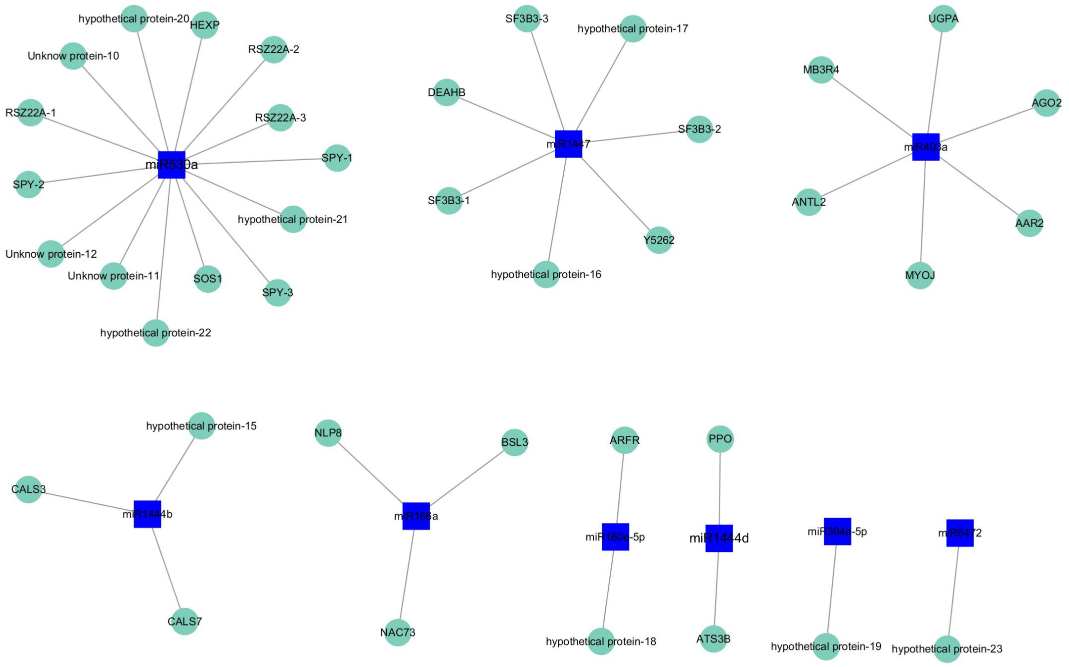

Supplementary Fig. S8 Networks of miRNAs and mRNAs in *P. qiongdaoensis*. Thirty-nine mRNAs were predicted as potential target genes of nine miRNAs, in the psRNATarget with an expectation ≤ 3.

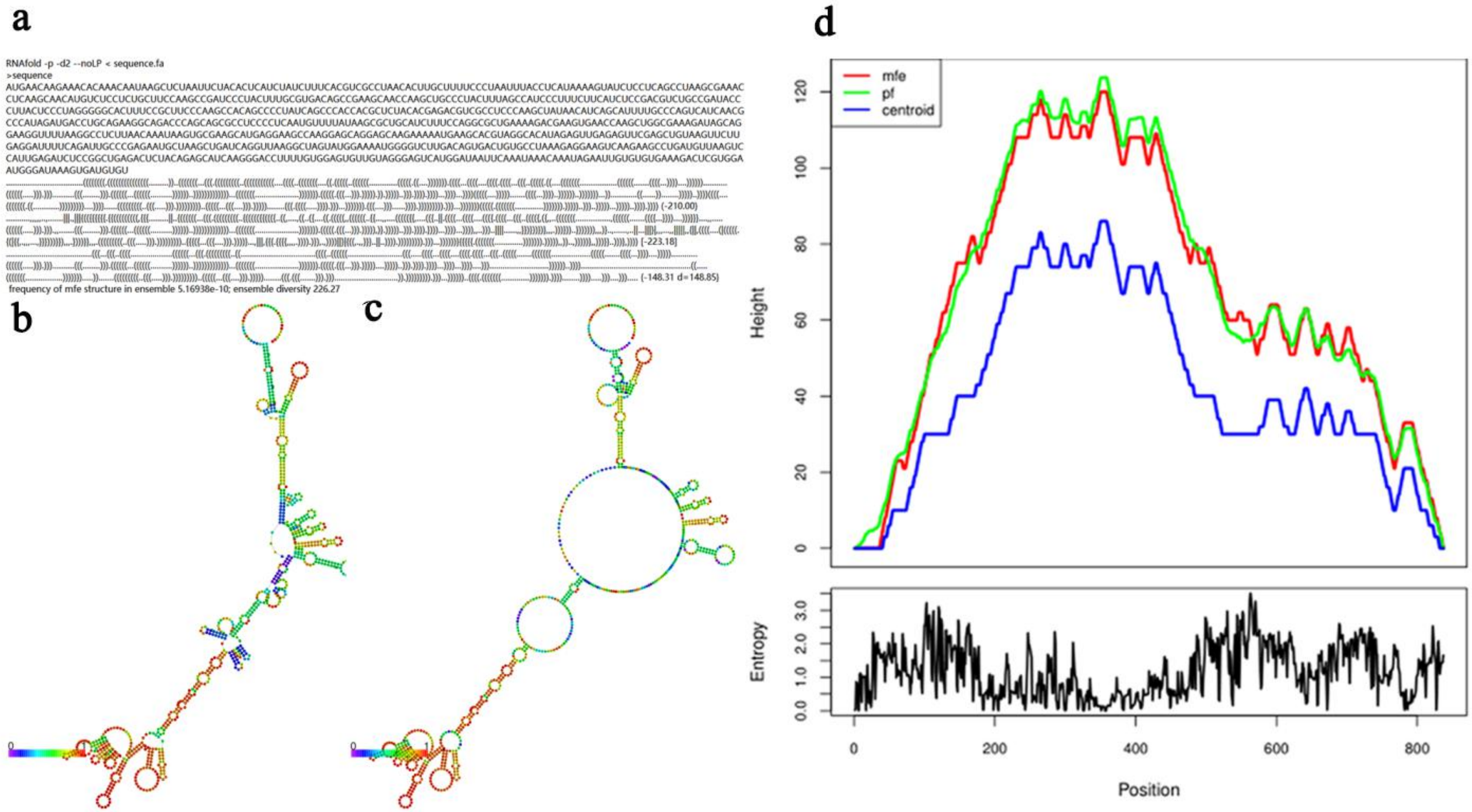

Supplementary Fig. S9 Prediction of lncHSP18.2 secondary structure. The optimal secondary structure in dot-bracket notation (a), minimum free energy secondary structure (b), centroid secondary structure (c), and mountain plot representation of the MFE structure,

thermodynamic ensemble of RNA structure, and the centroid structure (d). Base colors in the b and c diagrams represent base-pair probabilities. A mountain plot represents a secondary structure in a plot of height versus position, where the height m (k) is given by the number of bases at position k. The loops correspond to plateaus (hairpin loops are peaks), the helices to slopes.

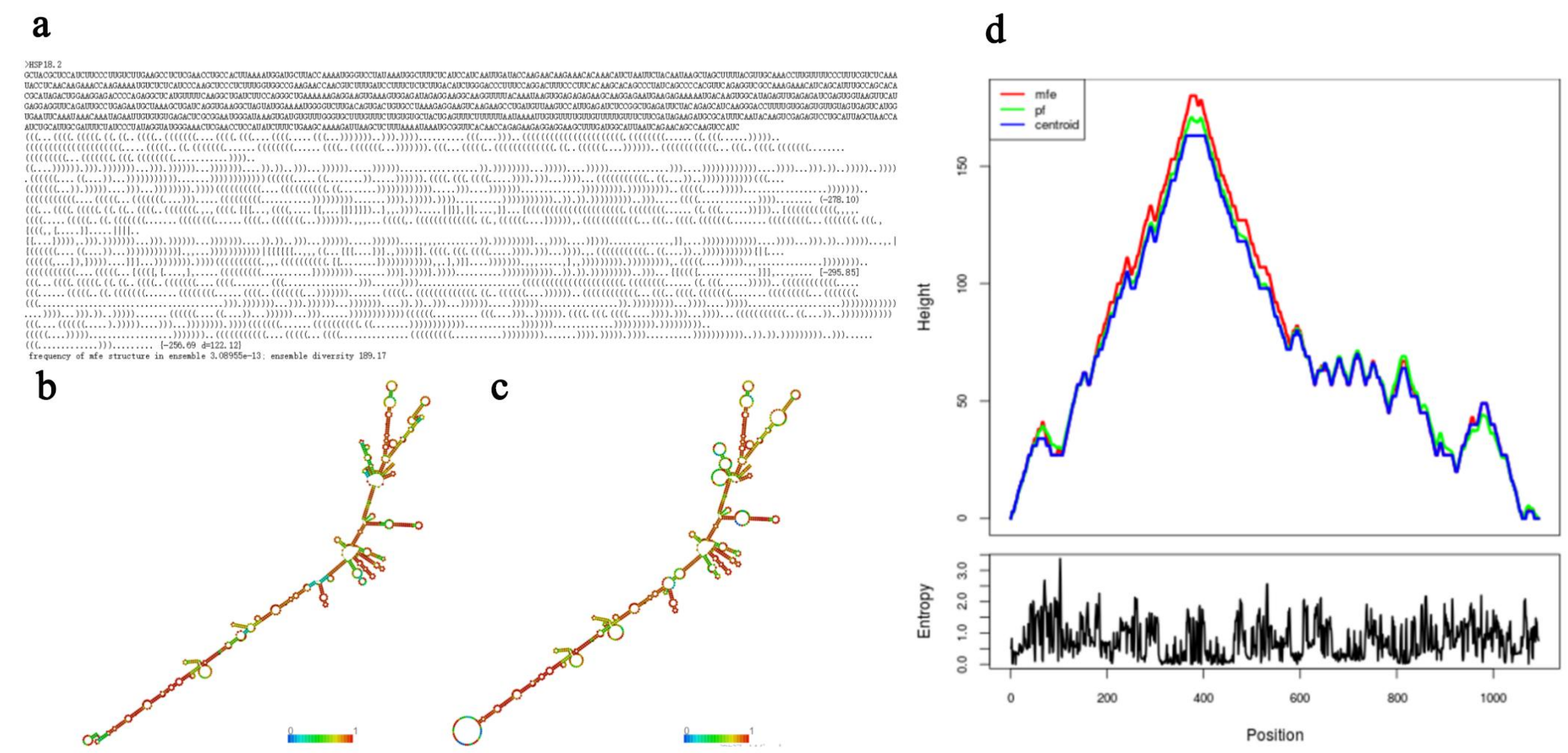

Supplementary Fig. S10 Prediction of mRNA (*HSP18.2*) secondary structure. The optimal secondary structure in dot-bracket notation (a), minimum free energy secondary structure (b), centroid secondary structure (c), and mountain plot representation of the MFE structure, thermodynamic ensemble of RNA structure, and the centroid structure (d). Base colors in the b and c diagrams represent base-pair probabilities. A mountain plot represents a secondary structure in a plot of height versus position, where the height

m (k) is given by the number of bases at position k. The loops correspond to plateaus (hairpin loops are peaks), the helices to slopes.
